# Tetanus in Diphtheria Antitoxin

**Published:** 1901-02-02

**Authors:** 


### TETANUS IN DIPHTHERIA ANTITOXIN.

The occurrence of an outbreak of tetanus among persons who had been treated for diphtheria by antidiphtheritic serum in Italy has naturally caused great alarm in that country, for there seems but little doubt that in some way or another a certain group of vials of serum sent out from the Institute in Milan had either become infected with the bacilli or contaminated with the toxin of tetanus. How this happened is as yet a mystery, but it appears that some twenty fatal cases have occurred, all of which were injected with serum obtained from the Sero-therapeutic Institute at Milan, and that the serum used in most of the fatal cases was derived from one batch of vials decanted from a much larger flask on a particular date. These were all separately sealed and numbered and sent out to the pharmacists, by whom they were distributed to the public in the same condition as that in which they were received. We have every reason to believe that in England the greatest care is taken, not only in the preparation of the serum, but in testing it before it is sent out, so as to be sure of the absence of pathogenic organisms. Certainly this is the case in regard to that which is prepared by the Royal Colleges, and there is no reason to believe that the private firms engaged in the same business are in any way behindhand in their efforts to ensure purity. At the same time we need not attempt to conceal the gravity of the fact that the increasing employment of powerful drugs, and of drugs as to the purity and strength of which the practitioner has no means whatever of judging, involves considerable risks, and we may add that it would be a great safeguard to the public and an unspeakable comfort to the medical profession, if all such substances could be issued under the sanction and authority of some officially appointed body.

So far the manufacture of antitoxins has fallen into but few hands, the Royal Colleges, the Jenner Institute, and a few leading druggists whose position gives a certain guarantee, and so far we have had every reason to be content—except, of course, in regard to price. But it must be confessed, as to the profession, that we do but buy a pig in a poke, and as to the patients that they take what is given them. Neither we nor they have the slightest means of ascertaining what is in the bottle in which we put such faith, and indeed, if we tried to put the matter to the test, the Vivisection Acts would entirely block the way and prevent our undertaking any investigations on the subject. Nor can we feel assured that the trade in antitoxins will always remain in the hands of trustworthy people. The prices paid are so very high, and the fabrication of sham sera would be so very simple, while the effectual testing of the finished product after it is once on the market would be so very difficult, that we can hardly hope that such a trade will long remain uncorrupted by undesirable business methods. We cannot then but think that the examination and standardising of these antitoxic sera before they are placed upon the market would be a work which might well receive the attention of the powers that be. If it is still thought absolutely necessary to maintain the present system of placing a delusive Government stamp upon quack medicines, the Government might well devote some of their ill-gotten gains from that source to the work of standardising and giving some form of official guarantee to medicines of well ascertained value.

